# Memory-like innate response to booster vaccination with MF-59 adjuvanted influenza vaccine in children

**DOI:** 10.1038/s41541-023-00702-1

**Published:** 2023-07-13

**Authors:** Dmitri Kazmin, Elizabeth A. Clutterbuck, Giorgio Napolitani, Amanda L. Wilkins, Andrea Tarlton, Amber J. Thompson, Emmanuele Montomoli, Guilia Lapini, Smiti Bihari, Rachel White, Claire Jones, Matthew D. Snape, Ushma Galal, Ly-Mee Yu, Rino Rappuoli, Giuseppe Del Giudice, Andrew J. Pollard, Bali Pulendran

**Affiliations:** 1 Institute for Immunology, Transplantation and Infection, Stanford University, Stanford, CA USA; 2 Oxford Vaccine Group, Department of Paediatrics, University of Oxford, and the NIHR Oxford Biomedical Research Centre, Oxford, UK; 3 Medical Research Council (MRC), Human Immunology Unit, University of Oxford, Oxford, UK; 4 The Royal Children’s Hospital Melbourne, Parkville, VIC Australia; 5 VisMederi Srl, Via Fiorentina, Siena, Italy; 6 Nuffield Department of Primary Care Health Sciences, Clinical Trials Unit, University of Oxford, Oxford, UK; 7 GlaxoSmithKline, Siena, Italy; 8 Department of Pathology, Emory University School of Medicine, Atlanta, GA USA; 9 Department of Pathology, and Microbiology & Immunology, Stanford University, Stanford, CA USA; 10 Emory Vaccine Center, Emory University, Atlanta, GA USA; 11 Present Address: Department of Molecular and Developmental Medicine, University of Siena, Siena, Italy; 12 Present Address: Fondazione Biotecnopolo, Siena, Italy

**Keywords:** Adjuvants, Protein vaccines

## Abstract

The pediatric population receives the majority of vaccines globally, yet there is a paucity of studies on the transcriptional response induced by immunization in this special population. In this study, we performed a systems-level analysis of immune responses to the trivalent inactivated influenza vaccine adjuvanted with MF-59 in children (15–24 months old) and in young, healthy adults. We analyzed transcriptional responses elicited by vaccination in peripheral blood, as well as cellular and antibody responses following primary and booster vaccinations. Our analysis revealed that primary vaccination induced a persistent transcriptional signature of innate immunity; booster vaccination induced a transcriptional signature of an enhanced memory-like innate response, which was consistent with enhanced activation of myeloid cells assessed by flow cytometry. Furthermore, we identified a transcriptional signature of type 1 interferon response post-booster vaccination and at baseline that was correlated with the local reactogenicity to vaccination and defined an early signature that correlated with the hemagglutinin antibody titers. These results highlight an adaptive behavior of the innate immune system in evoking a memory-like response to secondary vaccination and define molecular correlates of reactogenicity and immunogenicity in infants.

## Introduction

Seasonal influenza carries a significant health burden for young children^1,2^. Routine vaccination is used in some pediatric populations; however, the trivalent inactivated vaccine (TIV) has been shown to afford variable protection rates in infants and adults^3^. An oil-in-water adjuvant MF-59 is approved for use with TIV vaccine in adult populations and for children 6 months to 2 years of age in Canada. Adjuvanted TIV (ATIV) has been demonstrated to elicit stronger humoral response^4–11^ and to be more efficacious in early childhood than TIV^12,13^. The antibody response elicited by MF59-adjuvanted TIV vaccine is characterized by a higher magnitude and broader specificity than that induced by non-adjuvanted TIV^14^, and is mediated by a larger expansion of antigen-specific CD4^+^ T cells^14^. The increased efficacy and immunogenicity of MF59-adjuvanted vaccine may be explained by its positive effect on the recruitment of immune cells to the site of injection, higher antigen uptake by antigen-presenting cells, and more efficient priming of CD4^+^ T cells in draining lymph nodes, resulting in better antibody responses^15^. In addition, MF59 has been shown to boost innate and adaptive immune responses^16^, through a mechanism independent of the type 1 interferon signaling pathway^17^. MF59 also induces expression of multiple factors involved in immune cell recruitment from blood into the peripheral tissue^16–18^, and promotes differentiation of monocytes into immature dendritic cells^18,19^, resulting in increased antigen uptake and enhanced presentation^18,19^.

Systems immunology approaches have been used to understand the molecular mechanisms leading to the development of effective immune responses to a diverse spectrum of vaccines^20^, including yellow fever^21,22^, meningococcal vaccines^23^, shingles vaccines^24,25^, malarial vaccines^26,27^, mRNA vaccines^28^, and seasonal influenza vaccines in adult^29–33^ and in pediatric^6,34^ populations. Here we report the findings from a clinical study in which responses to MF59-adjuvanted TIV vaccine were compared between children (15–24 months old) and healthy young adults. This study was designed as a follow-up to a previous study, in which the responses to adjuvanted and non-adjuvanted TIV vaccines were studied in a pediatric population^6^. In that study, we observed that MF59-adjuvanted vaccine elicited a stronger transcriptional response in peripheral blood and was more immunogenic than the non-adjuvanted vaccine in terms of serological responses^6^. We also observed that the temporal dynamics of transcriptional responses in the peripheral blood of toddlers was altered by the addition of the MF59 adjuvant, making the responses more uniform across subjects and more similar to the time course of responses observed in adults immunized with TIV^6^. Therefore, this study was designed to address the following questions: first, what are the kinetics of transcriptional correlates of immunogenicity in young children following the prime and the boost immunizations with MF59-adjuvanted TIV vaccine; second, how do the responses to MF59-adjuvanted TIV compare between age groups; and, third, how do the responses to adjuvanted TIV in adults compare with those observed in multiple previous studies with non-adjuvanted TIV. We performed transcriptional profiling of the peripheral blood at various time points post prime and boost vaccinations, measured the gains in hemagglutinin inhibition (HAI) titers following the vaccination, and accessed cellular responses as they developed post prime and boost vaccinations. The data presented in this manuscript uncover the complex temporal dynamics of responses to adjuvanted TIV vaccine and shed light on the mechanism of action of MF59 adjuvant in infant and adult populations.

## Results

### Study design

**Fig. 1:**
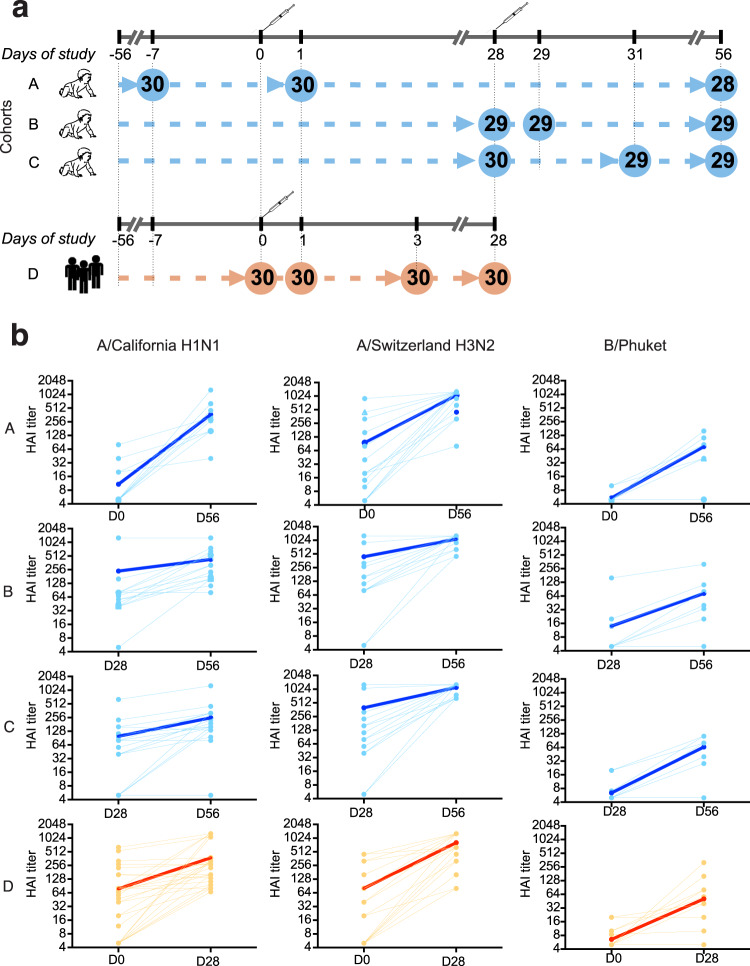
Study outline and serological responses. **a** Study outline. Ninety children, 15–24 months old, were randomized into three cohorts, A, B, and C, illustrated in blue color. Each infant received two injections of MF59-adjuvanted trivalent inactivated seasonal influenza vaccine, 28 days apart. Blood was sampled from each infant at three time points. Each infant from all three cohorts was sampled at day 56 of the study for the measurement of HAI titers. In addition, children in Cohort A were sampled at the pre-vaccination baseline (day −7 to day 0 of the study) and day 1 post prime vaccination. Children in Cohort B were sampled at day 28 (pre-boost baseline) and day 29 (1 day post boost vaccination). Children in Cohort C were sampled at day 28 and day 31 (pre-boost baseline and day 3 post boost vaccination, respectively). HAI titers were measured in all samples. Immunoprofiling of responding cellular subsets and transcriptional analysis was performed at the pre-vaccination baseline (Cohort A), day 1 post prime (Cohort A), Pre-boost baseline (Cohorts B and C), day 1 post boost (Cohort B) and day 3 post boost (Cohort C). In addition, 30 young (age 25–40 years) healthy adult volunteers received a single dose of the same vaccine (Cohort D, illustrated in orange color). Blood was sampled from all adult volunteers at the pre-vaccination baseline (day 28), day 29, and day 31 of the study (days 1 and 3 post vaccination), and day 56 of the study. HAI titers were measured at all time points, and immunoprofiling of responding cellular subsets and transcriptional analysis was performed at the baseline and days 1 and 3 post vaccination. **b** Serological responses. The changes in HAI titers against each one of the vaccine strains: H1N1 (A/California), H3N2 (A/Switzerland) and B (B/Phuket) are illustrated in each column. Each row corresponds to one of the four cohorts of the study. Each line represents a subject; bold lines represent mean changes in HAI titers across all subjects in a cohort.

The protocol for this study, NCT02529904, was ethically approved by NRES Committee South Central-Hampshire A, United Kingdom. Ninety children, aged 15–24 months, were randomized into three cohorts. Each participant received two doses of seasonal TIV vaccine with MF59 adjuvant, 28 days apart. Peripheral blood was collected as indicated in Fig. 1a. Due to the limitations on the number of blood draws that could be obtained from a child, we utilized a staggered sample collection design. Pre-vaccination baseline samples were obtained from individuals in cohort A. Pre-boost baseline samples were obtained from those in cohorts B and C. Children in Cohort A were sampled also at day 1 post primary vaccination. Children in cohorts B and C were sampled at day 1 and day 3 post boost vaccination, respectively. Blood was also collected from all subjects 28 days after the boost vaccination to access the serological responses. Thirty young (25–40 years of age) healthy adult volunteers (cohort D) received a single ATIV injection, and blood was collected from all adult subjects prior to vaccination and at days 1, 3, and 28 post vaccination (Fig. 1a).

### Reactogenicity and adverse effects

We assessed adverse effects on the day of injection and up to 3 days post vaccination. The ATIV vaccine was well tolerated, with no increase in irritability or fever among recipients in response to the primary vaccination, and a mild increase in irritability following the boost vaccination. Following the primary vaccination, the most common adverse effects were mild redness (19/90 or 21% of children), swelling (4/90, or 4.4%) and mild pain (29% of children). Redness and swelling were most observed either on the day of vaccination (day 0) or day 1 post primary vaccination, and in some cases lasted for more than 2 days (Supplementary Fig. 1A). Fever was not observed following the primary vaccination in children (Supplementary Fig. 1B). Following the boost, 24 out of 83 toddlers (29%) displayed mild redness, and 5 (6%)—mild swelling (Supplementary Fig. 1C). Swelling and redness commonly co-occurred in the same subjects (Supplementary Fig. 1). Additionally, 4% of children exhibited moderate pain, with a duration of 2 days or less (not shown). Five out of 83 (6%) child participants exhibited fever in response to the boost vaccination on the day of injection, which in two cases lasted for more than two days (Supplementary Fig. 1D). Among the adult participants 5/31 (16%) exhibited redness and 3/31 (10%)—swelling, which in some cases occurred in the same subjects (Supplementary Fig. 2A). Fever in response to vaccination was not observed among the adult participants (Supplementary Fig. 2B).

### Serological responses

Vaccine-specific antibody responses were assessed by hemagglutinin inhibition (HAI) assay. The results are illustrated in Fig. 1b. Both H1N1 (A/California) and H3N2 (A/Switzerland) strains proved to be equally immunogenic, and the resulting HAI titers at day 56 were not significantly different among the cohorts. Among children, we found greater prevalence of immunologically experienced subjects with respect to H3N2 strain (13/24) at baseline, compared with H1N1 (4/24). Most toddlers were naïve to the B strain (B/Phuket), which also was less immunogenic than the A strains. For children in cohort A seroconversion (4-fold increase in HAI titers) was achieved for 23/24 subjects for the H1N1 strain (A/California), 22/24 subjects for the H3N2 strain (A/Switzerland) and for 19/24 subjects for the B/Phuket strain. Seroprotection, as indicated by the HAI titer 1:629, estimated to correspond to a 90% protection rate^35^, was achieved for 6/24 subjects for the H1N1 strain, 20/24 subjects for the H3N2 strain, and no subjects achieved the HAI titer of 1:629 for the B/Phuket strain (Supplementary Fig. 3A). For the adults, seroconversion was achieved for 19/28 subjects for the H1N1 strain, 25/28 subjects for the H3N2 strain, and for 15/28 subjects for the B strain. Seroprotection (HAI titers of greater than 1:40) was achieved for all adult subjects for both A strains, and for 16/28 subjects for the B/Phuket strain (Supplementary Fig. 3B). The maximum gain in HAI titers (fold-change of day 56 to day 0) across all three vaccine strains was more likely to be achieved against the strain for which a subject was naïve at the baseline (Supplementary Fig. 3), consistent with the patterns shown in Fig. 1b.

### Transcriptional responses

**Fig. 2:**
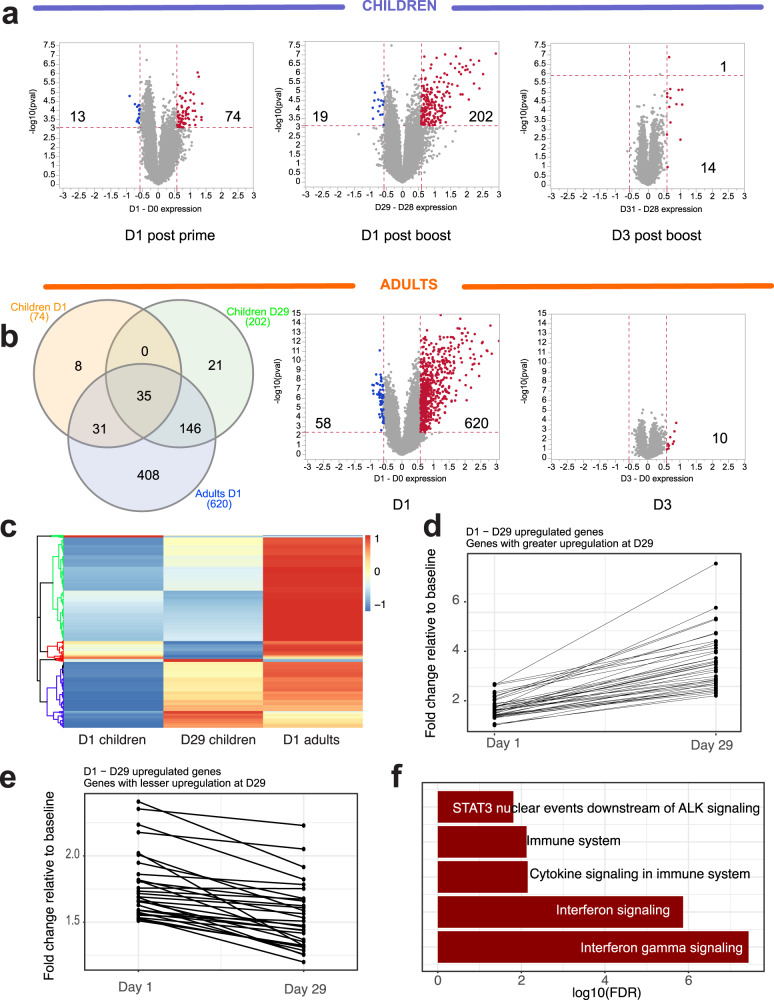
Transcriptional responses at gene level. **a** Volcano plots representing the quantitative transcriptional changes in peripheral blood occurring post vaccination. Each dot represents a probe set. *X* axis represents the log2 of the fold-change in gene expression relative to the corresponding baseline, *Y* axis represents -1*log10 of the corresponding *p* value (paired *t* test). Vertical red dashed lines are drawn at 1.5-fold change in gene expression. Horizontal red dashed lines are drawn at the *p* value corresponding to the FDR *Q* value of 0.05. Numbers represent the number of probe sets passing the above significance criteria. **b** A Venn diagram indicating overlaps among probe sets induced at day 1 post prime (D1), day 1 post boost (D29) in children and day 1 post injection in adults. **c** A heatmap illustrating patterns of regulation of all probe sets induced at D1 and D29 in children and D1 in adults. Fold-change values were standardized by row (z-score transformation). **d** Fold changes of genes with greater upregulation at D29 than at D1. For this plot, 39 probe sets that are upregulated at D29 by 2-fold more than they are upregulated at D1 were selected. **e** Fold changes of genes with lesser upregulation at D29 than at D1. For this plot, 30 probe sets that are upregulated at D29 by 2-fold less than they are upregulated at D1 were selected. **f** Reactome pathway enrichment of 39 probe sets shown in panel D. These are the genes that are upregulated more strongly in response to the secondary vaccination compared to the primary.

**Fig. 3:**
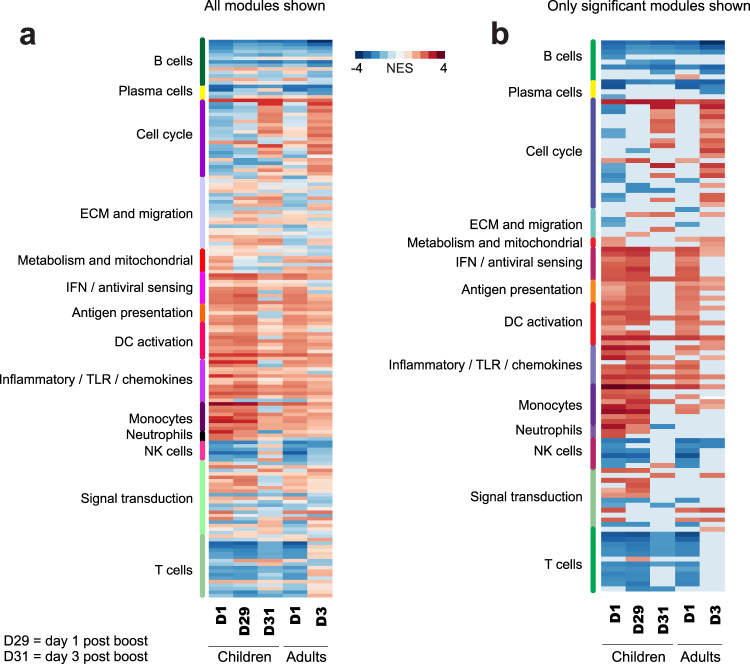
Transcriptional responses at module level. **a** Qualitative analysis of changes in gene expression by gene set enrichment analysis (GSEA). Each row represents a gene module, each column—a time point. Color represents the normalized enrichment score. Modules are grouped according to the high level annotation category, as indicated by the legend on the left of the heatmap. All modules are shown, regardless of the statistical significance of enrichment. **b** Same as (**a**), showing only modules with statistically significant (FDR < 0.05) enrichment. Non-significant modules are shown as blank.

We observed robust transcriptional responses in peripheral blood at day 1 post prime and post boost in toddlers. Post-boost responses were generally stronger, with a total of 221 genes passing the significance cutoffs (FDR < 0.05 and fold-change relative to baseline greater than 1.5-fold). Day 3 responses were very weak, consistent with the previously observed trend^6^ (Fig. 2a). Responses in adults were stronger than in children, with a total of 678 up- or down-regulated genes satisfying the significance criteria. Similar to the responses observed in young children, responses at day 3 in adults were very weak, with no significant genes detected (Fig. 2a). Among the upregulated genes, out of 74 probe sets significantly upregulated at D1 in children, 35 were found to be also upregulated at D29, while 39 were unique to D1 (Fig. 2b, c). The 35 probe sets (reporting on 23 unique named genes) that are upregulated at both D1 and D29 are significantly enriched in Reactome pathways relevant to interferon signaling, such as interferon gamma signaling (FDR = 8.8×10^–9^) and interferon signaling (FDR = 3.38×10^–7^). The 39 probe sets unique to D1 (the red cluster in Fig. 2c) were not significantly enriched in any immunologically relevant pathway. The majority of genes upregulated at D1 or D29 in children were also upregulated in adults (Fig. 2b, c), and with greater magnitude (Fig. 2c), and the 620 probe sets upregulated in adults were strongly enriched in the interferon signaling pathway (FDR = 1.98×10^–12^). Many genes whose expression was induced at D1 post prime vaccination were also induced at D1 post boost (D29), but to a greater magnitude (Fig. 2d). Reactome pathway overrepresentation analysis indicated that these genes are strongly enriched in interferon signaling pathways (Fig. 2f). In contrast, genes whose upregulation at D29 was less than upregulation at D1 (Fig. 2e) were not enriched in any immunologically relevant pathway (data not shown). Functional responses to vaccination were assessed by gene set enrichment analysis (GSEA) using blood transcriptional modules (BTMs)^23^. We observed strong activation of multiple modules relevant to innate immunity at days 1 post prime and post boost vaccination in children, which diminished at day 3 post boost (Fig. 3). These include modules in interferon/antiviral sensing, antigen presentation, DC activation, inflammatory/TLR/chemokines, monocytes and neutrophils groups. In contrast, NK cell modules were strongly suppressed at day 1 post prime and day 1 post boost. Cell cycle modules were strongly upregulated at day 3 post boost. Among the modules relevant to the adaptive immunity, we observed strong suppression of B cell modules and plasma cell modules at all time points, and strong suppression of multiple T cell modules at day 1 post prime and day 1 post boost. This suppression was only partially relieved at day 3 post boost in children (Fig. 3). Transcriptional responses in adults generally mirrored those observed in children. One notable difference was the reversal of suppression of T cell modules at day 3 post vaccination. At day 3 post boost in children these modules continue to be suppressed, while in adults we observed a mild (statistically insignificant) upregulation (Fig. 3 and Supplementary Fig. 4).

### Transcriptional signatures at baselines prior to primary and booster vaccinations

**Fig. 4:**
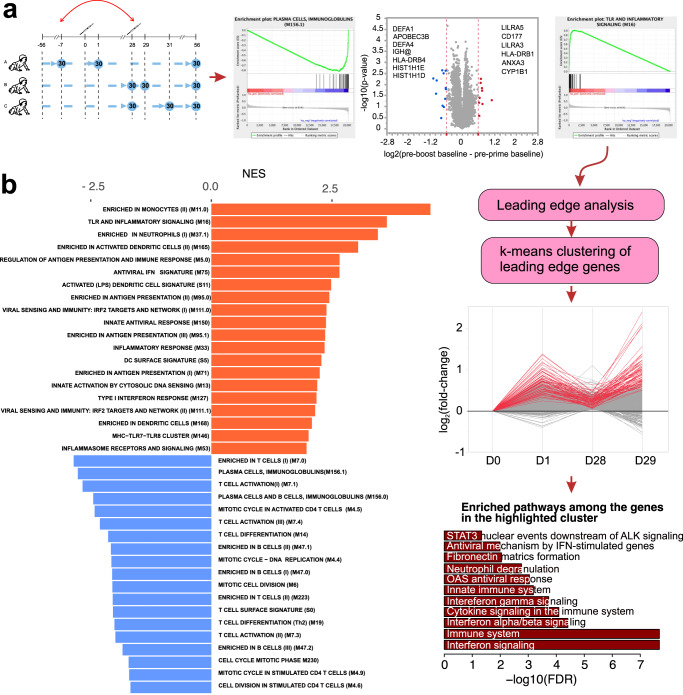
Comparison of pre-prime (Cohort A) and pre-boosts (Cohorts B and C) baselines. **a** Volcano plot is as described above. No probe sets passed the FDR < 0.05 filter. Panels on the left and right of the volcano plot illustrate the enrichment of select gene sets in the list of genes ordered by the difference in gene expression at day 28 (visit 4, pre-boost baseline) compared to day 0 (pre-vaccination baseline). Genes were ranked according to the fold-change between the D28 and D0 baselines and GSEA was performed on the ranked gene list, followed by the leading edge analysis to identify genes, whose expression is most strongly perturbed between the two baselines. Expression of the leading edge genes were analyzed by *k*-means clustering (*k* = 3), The cluster of genes that are upregulated at D1, D28 and D29 is highlighted in red in the line plot. Reactome pathway enrichment analysis was performed on genes within this cluster, and illustrated on the bottom panel. **b** Representative enriched modules from the GSEA analysis of the ranked list of genes.

Direct comparison of pre-prime and pre-boost baselines yielded no significantly up- or down-regulated genes at day 28 compared with day 0 (Fig. 4a). However, gene set enrichment analysis (GSEA)^36^ performed on lists of genes ranked by the relative fold-change in expression at day 28 compared with day 0, indicated that many gene modules relevant to adaptive responses (T cell and plasma cell modules), as well as NK cell modules, appear to be more strongly expressed at day 0 compared with day 28, indicating their suppression at the pre-boost baseline (Fig. 4a, b). In contrast, many modules relevant to the innate responses were positively enriched at day 28 baseline, indicating continuous activation of innate responses between the pre-prime and pre-boost time point (day 28 vs day 0) (Fig. 4a, b). We performed a leading-edge analysis of innate immunity modules with significant positive enrichment at the pre-boost baseline and traced the activation pattern of these genes across the timeline of this study. *K*-means clustering of expression patterns of these genes indicated several distinct patterns. One cluster (indicated in red on the line plot in Fig. 4a) contained genes, whose expression is induced at day 1 post prime, and does not return to baseline 28 days after the first vaccination. After the boost injection, these genes are induced again, and in many cases, to a greater magnitude than at day 1 post prime (Fig. 4a). Reactome pathway analysis of genes within this cluster revealed strong enrichment of interferon signaling and antiviral response pathways (Fig. 4a, bottom).

### Transcriptional correlates of reactogenicity

**Fig. 5:**
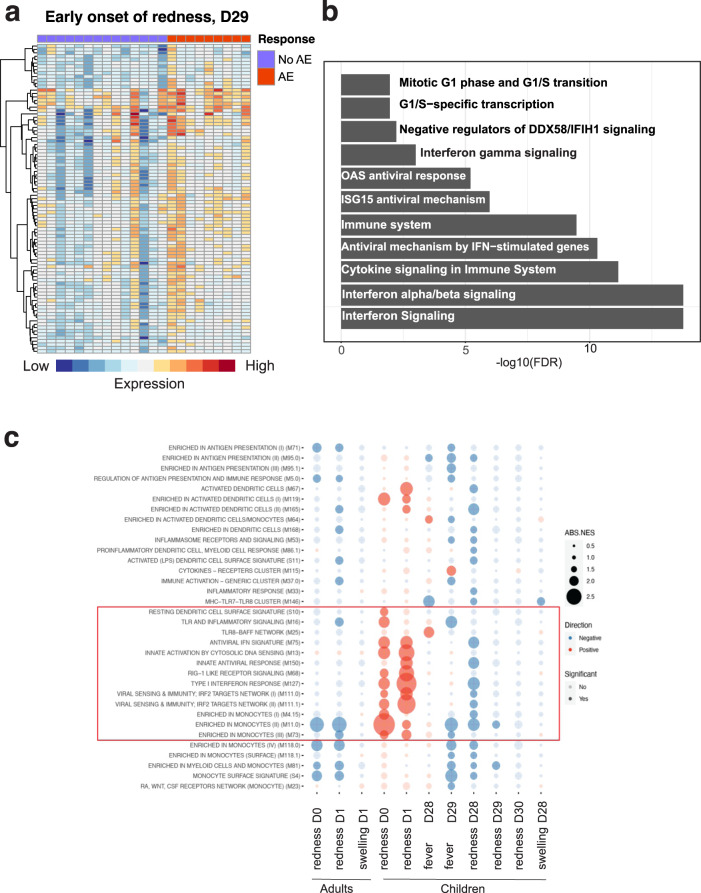
Transcriptional correlates of reactogenicity. **a** A heatmap showing genes, whose expression tracks with early onset of erythema in toddlers post secondary vaccination. Genes were selected as having baseline-normalized expression level in responders more than 2-fold higher than in non-responders. Early onset is defined as erythema larger than 1 mm observed no later than 24 h post injection. **b** Reactome pathway analysis of the genes presented in (**a**), horizontal axis represents -log_10_(FDR) for enrichment of a given pathway. Only pathways that are statistically significantly enriched are shown. Significant enrichment is defined as FDR < 0.05. **c** Baseline correlates of reactogenicity in children and adults, as defined by gene set enrichment analysis. Red color of circles indicates positive correlation with the occurrence of adverse effects, size indicates the absolute value of the normalized enrichment score (ABS.NES).

We investigated the transcriptional and cellular correlates of vaccine reactogenicity, as measured by fever, redness and swelling, in toddlers and adults. Appearance of redness and/or swelling at the site of the injection of any measurable size (>1 mm) was considered an adverse effect. For fever, an increase of body temperature post vaccination by more than 0.5 °C compared to the pre-vaccination baseline, combined with measured temperature post vaccination of greater than 37 °C was considered an adverse effect. We found no transcriptional or cellular correlates of fever. However, we identified a large set of genes, whose expression levels after the secondary vaccination relative to the pre-boost baseline discriminates toddlers who displayed early onset of erythema (1–24 h) from those who did not (Fig. 5a). When we investigated pathway enrichment in this set of genes using Reactome database (www.reactome.org), we noted a strong prevalence of multiple pathways relevant to interferon response (Fig. 5b). Likewise, we identified multiple interferon response pathways as enriched among the genes that are more strongly upregulated in toddlers, who displayed an early onset of swelling at the site of injection following the secondary vaccination, compared to toddlers that did not display swelling (Supplementary Fig. 5A). Thus, we identified an elevated interferon response to the boost vaccination in peripheral blood as a correlate of early onset of local reactogenicity in toddlers. We did not observe a consistent pattern of transcriptional correlates of local reactogenicity in toddlers following the prime vaccination. In adults, we did not observe transcriptional correlates of early onset of erythema; however, we did observe that elevated expression of multiple genes, relevant to interferon response in adults subjects that displayed an early onset of swelling (Supplementary Fig. 5B). Finally, we also observed that multiple interferon response pathways are enriched among the genes more highly expressed in adults that displayed a late (24–72 h) onset of erythema (Supplementary Fig. 5C). Taken together, these data allow us to conclude that systemic upregulation of interferon response, observed in peripheral blood, is a hallmark of concomitant local reactogenicity of the vaccine, but not of systemic response, such as fever.

We then investigated whether there are transcriptional features existing at the baseline (prior to vaccination) that track with increased reactogenicity post vaccination. We found that multiple modules related to interferon signaling and monocytes represent positive baseline correlates of immediate (the day of vaccination) and early (day 1 post vaccination) onset of erythema post prime vaccination in children, but not in adults (Fig. 5c). These data demonstrate that activation of interferon response-related modules at baseline pre-vaccination is a marker of vaccine reactogenicity post vaccination.

### Transcriptional correlates of immunogenicity

**Fig. 6:**
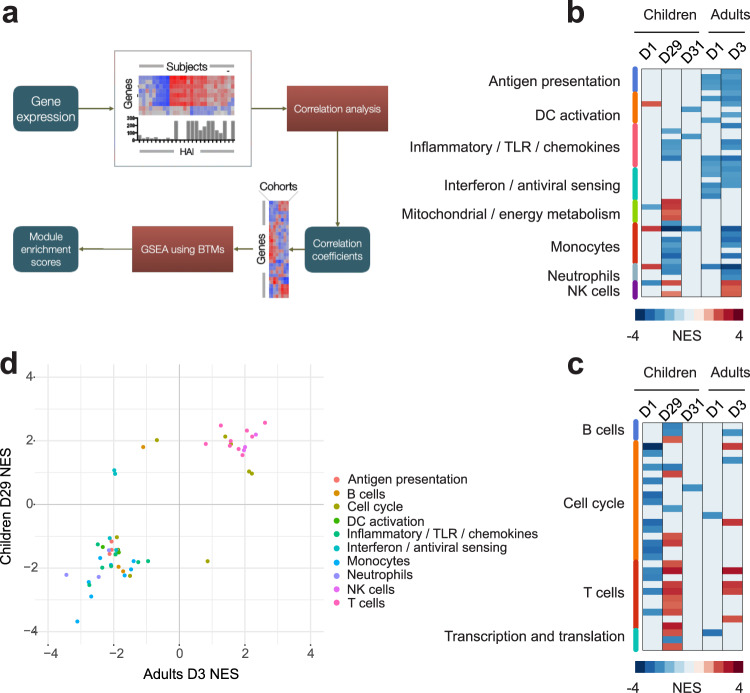
Transcriptional correlates of antibody responses. **a** The schematic of the methods used to generate the transcriptional correlates of immunogenicity. For each gene correlation with HAI titers was calculated, and genes were ranked based on the correlation coefficients. Gene set enrichment analysis was performed on the ranked list of genes and the enrichment scores for each gene modules were plotted in (**b**, **c**). **b**, **c** Significantly enriched modules from the innate immunity (**b**) and adaptive immunity (**c**) groups. Each row represents a module. Non-significantly (FDR > 0.05) enriched modules are shown as blank. **d** Scatterplot illustrating the similarity between the correlates of HAI responses for children at D29 and adults at D3. Each dot represents a module.

We have utilized GSEA in combination with correlation analysis to identify gene modules whose up- or down-regulation most strongly correlates with the downstream accumulation of vaccine-specific antibodies (Fig. 6a). We have observed that the induction of multiple innate immunity gene modules involved in antigen presentation pathways, DC activation, monocytes, inflammatory/TLR/chemokines, at day 1 post primary vaccination, were positively correlated with downstream HAI titers (Supplementary Fig. 6A), although for most of these modules the enrichment did not reach statistical significance (Fig. 6b). At the same time, most adaptive immunity modules, including cell cycle and T cell modules, at day 1 post prime, were negatively associated with HAI titers (Fig. 6c and Supplementary Fig. 6B). Transcriptional correlates of HAI responses at D1 post boost (D29 of the study) were very different from those correlates observed at D1 post prime. These correlates included negative association of several innate immunity modules (Fig. 6b), while multiple T cell modules and cell cycle modules showed significant positive association with downstream serological responses (Fig. 6c). At day 3 post boost very few significant associations were observed in children (Fig. 6b, c). Transcriptional correlates of immunogenicity in adults at day 1 were weak and mostly included negative correlations for innate immunity modules, like the correlates observed at day 29 in children (Fig. 6b). Positive association of T cell modules, observed in children at day 1 post boost was not observed in adults at day 1 (Fig. 6c). However, correlates observed in adults at day 3 post vaccination are strikingly similar to the correlates observed in children at day 1 post boost, with negative associations of multiple innate immunity modules, and positive correlations observed for T cell modules and NK cell modules (Fig. 6b–d and Supplementary Fig. 6A, B).

### Enhanced activation of myeloid cells following booster vaccination

**Fig. 7:**
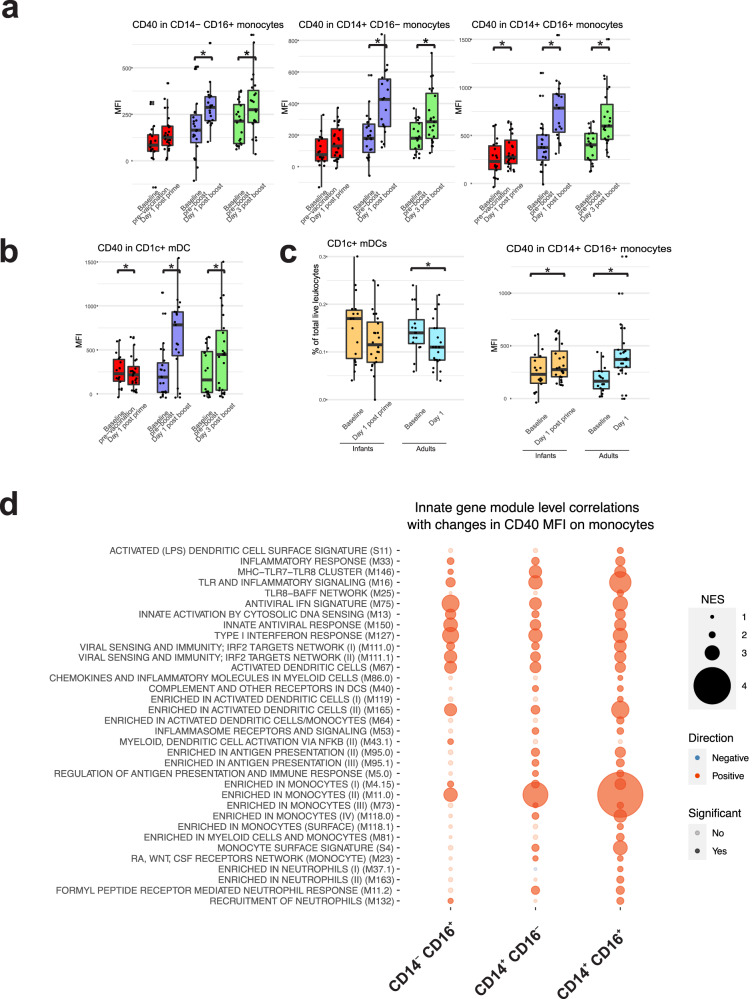
Cellular responses. Overall changes in the frequency of select cellular populations are shown for the infant cohorts (**a**, **b**), and directly comparing responses observed in adults and children (**c**). Asterisks indicate significant differences based on paired Wilcoxon test. For panels A and B, the changes occurring at day 1 post prime are shown in red; at day 1 post boost – in blue, and at day 3 post boost – in green. For (**c**), changes occurring in children are shown in orange, and in adults – in cyan. Horizontal lines indicate the median; boxes extend from first to third quartiles (25th and 75th percentiles); whiskers extend to the most distant value within 1.5 of interquartile range (IQR). **d** Correlations between transcriptional responses to boost vaccination and activation of monocyte subsets. Changes in gene expression between day 28 (pre-boost baseline) and day 29 (day 1 post boost) were correlated with the changes in CD40 MFI on monocyte subsets for the same days. Genes were ranked by correlation coefficients and GSEA was performed on the ranked lists of genes using BTMs. Size of circles represents the magnitude of enrichment, color – the direction of enrichment; opaque circles indicate the lack of statistical significance of enrichment.

The transcriptional analysis described here and in the previous study^6^ suggest a correlation between multiple innate immunity transcriptional modules and antibody responses. To confirm and extend this observation, we determined if these transcriptional signatures were associated with changes in the frequency and the activation profile of distinct myeloid cell populations. Thus, we analyzed multicolor flow cytometry data generated from the analysis of fresh blood samples collected over the course of the study, and quantified neutrophils, basophils, eosinophils, CD1c^+^ myeloid cDC2, CD141^+^ myeloid cDC1, CD123^+^ plasmаcytoid DCs, as well as classical (CD14^high^CD16^-^), intermediate (CD14^high^CD16^+^) and non-classical (CD14^low^CD16^high^) monocytes. The gating strategy is presented in Supplementary Fig. 7. Among monocyte subpopulations, only classical CD14^high^CD16^-^ monocytes showed a slight increase at day 3 post boost (data not shown). We observed, however, a strong and significant increase in the expression of the activation marker CD40 on all monocyte subsets at days 1 and 3 post boost, as well as at day 1 post prime (CD14^high^CD16^+^ intermediate monocytes only) (Fig. 7a). Strikingly, the increase in CD40 on monocyte subsets was most pronounced at days 1 or 3 post boost relative to the increase post prime (Fig. 7a), suggesting an enhanced innate response to secondary vaccination, consistent with the enhanced transcriptional signatures of innate immunity during the secondary vaccination (Figs. 2 and 3). Consistent with this, we also observed a significant increase in the expression of CD40 on CD1c^+^ mDCs at days 1 and 3 post boost (Fig. 7b). The changes in cell frequencies and CD40 expression observed in children were recapitulated in the adult cohort (Fig. 7a, c). Enhanced activation of myeloid cells following the boost vaccination is consistent with the enhanced activation of multiple innate immunity pathways (Fig. 2d, f). We performed a gene-level correlation analysis to link the differences in gene expression between the pre-boost baseline and day 1 post boost and the changes in CD40 MFI at the same time points, followed by the GSEA analysis of genes ranked by their correlation coefficients. We observed that the expansion of a large number of innate immunity modules at day 1 post boost positively correlates with the increase of CD40 expression on all three monocyte subsets (Fig. 7d). Notably, many of the modules whose expression correlates with increased CD40 MFI also show persistent upregulation at the pre-boost baseline (day 28) compared to the pre-prime baseline (day 0) (Fig. 4b).

## Discussion

**Fig. 8:**
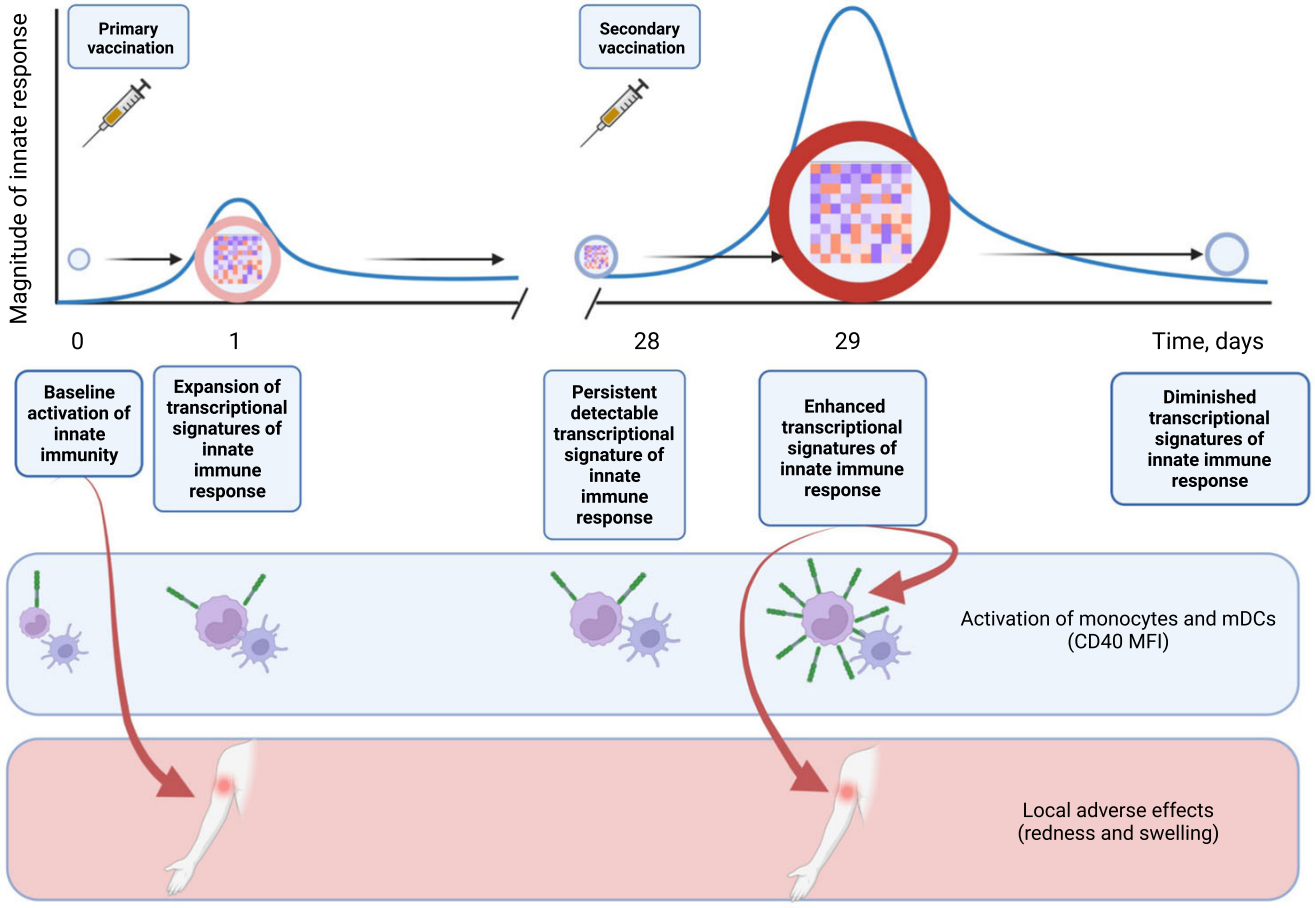
Mechanistic summary. Primary vaccination triggers a transient peak of innate immune response, as evidenced by transcriptional analysis. This innate response, however, persists at low, but detectable level for 28 days post primary vaccination and does not diminish to the pre-vaccination baseline. Following the secondary injection, innate immune response is amplified compared to the same response to the primary vaccination. Transcriptional innate signatures of immune response correlate with elevated activation of monocytes and dendritic cells, as well with local adverse reactions to the vaccine. Created with BioRender.com.

In this study, we are describing a memory-like behavior of innate immune system, which becomes primed by the initial vaccination and responds more robustly to the secondary vaccination 28 days later (Fig. 8). These enhanced responses are evidenced by both more robust induction of transcriptional signatures of innate immunity, primarily interferon response pathways, and elevated activation of monocytes and dendritic cells after the secondary vaccination compared to the responses observed after the primary (Fig. 8). The same interferon response pathways that, on a transcriptional level, persist for 28 days after the primary vaccination and respond more robustly after the secondary, are also shown to be the correlates of local adverse effects of the vaccine, both at the baseline and post vaccination (Fig. 8).

In the current study, we observed elevated innate responses following the boost vaccination, compared to the prime immunization. Boost immunization resulted in a stronger transcriptional response, with nearly three times the number of upregulated genes, compared with the prime vaccination (Fig. 2a). Among the genes upregulated at D1 post prime vaccination, roughly half were also induced at D1 post boost, while the rest were specific to the post-prime time point (Fig. 2b, c). Of interest, those genes that were consistently upregulated both post prime and post boost, were strongly enriched in interferon signaling pathways, while genes, specific to the post-prime responses were not. Quantitatively, many genes upregulated at D1 post prime were upregulated with a greater magnitude post boost (Fig. 2c, d). These genes were strongly enriched in interferon signaling pathways (Fig. 2f). Consistent with these observations, we observed enhanced activation of monocyte subsets and myeloid DCs in response to secondary vaccination, relative to that observed after primary vaccination (Figs. 7a, b and 8).

It is generally postulated that innate immunity does not possess a memory component, and cells that mediate the initial response to the pathogen do so in a naïve fashion each time they encounter the same antigen. However, recently a concept of trained innate immunity^37,38^ has been proposed, that posits that in some settings innate immune responses can be primed by the initial exposure to respond more robustly to re-challenge^39^. This priming is mediated by epigenetic reprogramming of responding innate cellular populations, such as monocytes^40^. Our recent work shows that adjuvants elicit such priming of the innate immune system, resulting in enhanced innate responses upon re-challenge^41^. So, AS03 adjuvant, administered with H5N1 pandemic vaccine was shown to stimulate persistent epigenetic remodeling, increasing accessibility of multiple interferon response loci, resulting in increased expression of genes involved in antiviral response, and elevated resistance to unrelated viruses^41^. Epigenetic priming of the innate immune system can be long lasting. So, administration of OVA antigen in mice in combination with 3M-052-Alum adjuvant resulted in persistent changes in innate immune cells that lasted for at least 28 days^42^. Consistent with this notion, our recent work suggests that secondary vaccination with the BNT162b2 COVID-19 mRNA vaccine also induces an enhanced secondary innate response, characterized by an enhanced transcriptional signature of IFN signaling^28^. This was recently confirmed in other studies^43^.

An inclusion of both pre-prime and pre-boost baselines allowed us to assess the long-lasting transcriptional responses to the prime vaccination in peripheral blood. Of interest, we observed that multiple innate immunity modules were upregulated at the day 28 pre-boost baseline compared with day 0 pre-prime baseline, suggesting their continuous activation as late as 28 days post prime vaccination (Figs. 4b and 8). This is similar to the re-activation of multiple innate immunity modules observed by us with an unrelated RTS,S malarial vaccine, which occurred 28 days following the previous vaccinations^26^. Long term effects of vaccines on innate immunity have been previously described. In particular, a yellow fever YF17D vaccine is known to induce long-term activation of monocytes and NK cells, lasting up to 60 days post vaccination^44–46^. Trivalent inactivated influenza vaccine was previously shown to induce long-lasting increase in frequency of both classical and inflammatory monocytes producing inflammatory cytokines IL-6, IL-10 and TNFα, which peaked at 28 days post vaccination^47^. Frequency of TNFα producing inflammatory monocytes at 28 days post vaccination was also linked to vaccine responsiveness in young volunteers^47^. In this study we observed strong induction of a large number of innate immunity gene modules at D28 post prime vaccination compared to the D0 pre-prime baseline (Fig. 4b). We also observed that a set of genes with high relevance to the interferon response is strongly induced at D1 post prime, but their expression does not return to baseline 28 days later. Following the boost, these same genes become re-activated again, and in some cases, their upregulation post boost exceeds in magnitude their upregulation post prime (Fig. 4a). Together with the published data, our results indicate that immune responses to the prime vaccination persist for at least 28 days, and that the immune system does not return to the steady state between prime and boost vaccine administrations, instead existing in the “heightened preparedness” state and responds more robustly to the boost injection. This difference in baselines is important for interpretation of the results presented here, since cohorts B and C, sampled at day 1 and day 3 post boost, lack the pre-vaccination baseline, and the post-vaccination responses are compared to the pre-boost baselines for the respective subjects.

We demonstrated that concomitant activation of transcriptional interferon response program tracks with local vaccine reactogenicity, such as redness at the site of injection (Fig. 5a, b, Supplementary Fig. 5C and Fig. 8), and swelling (Supplementary Fig. 5A, B) in both children and adults. Interferon signaling as a trigger of local reactogenicity has been previously described for the BNT162b2 COVID-19 vaccine^48^. This is a novel concept, as previously it was generally postulated that local production of cytokines, vasodilators, prostaglandins and complement factors, in combination with local cell recruitment, triggers local adverse effects, such as pain, redness and swelling^49^. MF-59 was previously shown to elicit distinct physiological response patterns, which were not observed with unadjuvanted TIV vaccine^50^. It was also noted that elevated inflammatory responses, typical for adjuvanted vaccines^50–52^ tracks with increased reactogenicity, although no direct mechanistic link was proposed^50^. We also investigated baseline correlates of downstream vaccine reactogenicity and demonstrated that activation of multiple innate immunity gene modules, including IFN signaling at baseline tracks with local reactogenicity in children (Figs. 5c and 8). To our knowledge, this is the first demonstration that activation of IFN signaling pathways prior to vaccination correlates with local adverse effects of the vaccine. Of interest, we did not identify any transcriptional signatures of systemic reactogenicity, such as fever.

Taken together, our observations highlight important differences transcriptional and cellular responses to the prime vaccination (as the majority of young children are naïve to at least one vaccine strain) and recall responses to boost vaccination in children or vaccination in adults. Presented data highlight the differences and similarities of the mechanisms that lead to the development of protective immunity to the adjuvanted TIV vaccine in children and adults and affords a glance at the dynamic picture of how these mechanisms unfold following the naïve and recall responses.

## Methods

### Sample collection and processing

Blood for transcriptomics, HAI and cellular response was collected at all of the time points indicated in Fig. 1a.

#### Transcriptomics

From all cohorts, 0.5–1.0 ml blood was obtained in 2.76 ml of Paxgene buffer (PreAnalyx, UK), and gently inverted to mix. The blood was kept at room temperature until storage at −80 °C (within 2–6 h of venipuncture) until being shipped for RNA isolation, hybridization and analysis at Emory University (Atlanta, GA).

#### Plasma collection for hemagglutination (HAI) analysis

Heparinized whole blood (0.5–3.5 ml) was collected from all participants, stored at room temperature prior to plasma separation within 6 h of venipuncture. Blood was centrifuged at 1800 × *g* for 10 min, the plasma layer transferred to a fresh tube and re-spun at 1800 × *g* for 10 min to remove residual cells and then aliquoted and stored at −80 °C prior to shipment for HAI analysis, to VisMederi Srl, Sienna, Italy.

#### Isolation of peripheral blood mononuclear cells (PBMCs)

Heparinized whole blood (1–5.0 ml) was collected from all participants and stored at room temperature for shipment to the Weatherall Institute for Molecular Medicine, Oxford, UK for processing and analysis of cellular responses.

### Hemagglutination (HAI) assays

VisMederi Srl Laboratories (Sienna, Italy) conducted the HAI assays in agreement with standard procedures. The assays were conducted using viral antigens from influenza strains (A/California/07/2009, A/Switzerland/9715293/2013, and B/Phuket/3073/2013) provided by the National Institute for Biological Standards and Control (NIBSC). HAI was measured at baseline for each vaccine dose (cohort A day 0, cohort B and C day 28) and 28 days post-second vaccination for children; and baseline and 28 days post-vaccination in adults. Samples were analyzed in duplicate and treated to remove non-specific inhibitors of HAI. Twofold, serial dilutions, starting at 1:10 were incubated with the same volume of influenza antigen, followed by incubation with turkey red blood cells. The level of hemagglutination was assessed by the naked eye and HAI titers calculated as the reciprocal value of the highest serum dilution where hemagglutination was still inhibited. Seroprotection rates were calculated at HAI thresholds of 1:40 for adults and 1:629 for children, as previously described by Black et al.^35^ as a more accurate correlate of protection for children. The fold rise from baseline to day 28 (adults and children) and to day 56 (children only) was used for seroconversion rates.

### RNA isolation and hybridization

Total RNA was isolated from whole blood. Transcriptional profiling was performed using Affymetrix (Santa Clara, CA) HG-133plus2.0PM chips.

### Transcriptional data analysis

Raw hybridization data were QCed using ArrayQualityMetrics R package^53^. Intensity data were normalized by RMA. Downstream processing was performed using custom codes written in R.

### Statistical methods

For differential gene expression, paired *t* test was used, followed by false discovery rate (FDR) adjustment at α level 0.05. Significantly regulated genes were defined as those with FDR < 0.05 and absolute fold change >1.5 (0.585 on a log2 scale). Reactome pathway analysis was performed online (www.reactome.org). Gene set enrichment analysis was performed using GSEA version 3.0 with 5,000 permutations. Significance of module enrichment was determined by FDR *q* value. *K*-means clustering was performed in SAS JMP Pro 16 software. For the genes whose expression tracks with adverse effects, genes were selected based on greater than twofold mean difference in baseline-adjusted expression levels between AE and no AE groups. For correlations with HAI titers, gene-wise baseline-adjusted expression levels were correlated to HAI titers and Spearman Rho coefficients were used as input to GSEA. For cellular responses, significant differences in cell frequencies were determined by paired Wilcoxon test.

### Analysis of cellular responses

Whole blood was processed and stained within 4 h from collection. 1 ml of blood was incubated for 15 min on ice with ACK buffer to lyse red blood cell. Cells where extensively washed in FACS Buffer containing 2% Fetal Bovine Serum (Sigma) and 0.05% NaN3 (Sigma) and then stained with a cocktail of antibodies directed against CD33 (WM53, PE, eBioscience, catalog number 12-0338-42, dilution 1:40), CD123 (6H6, PE-e610, eBioscience, catalog number 61-1239-42, dilution 1:40), CD1c (L161, PE-Cy7, Biolegend, catalog number 331515, dilution 1:20), CD16 (eBioCB16, APC, eBioscience, catalog number 17-0168-42, dilution 1:40), CD11b (M1/70, A700, Biolegend, catalog number 101222, dilution 1:10), HLA-DR (L243, APC-Cy7, Biolegend, catalog number 307617, dilution 1:40), CD80 (2D10, Bv421, Biolegend, catalog number 305221, dilution 1:20), CD14 (M5E2, BV605, Biolegend, catalog number 301833, dilution 1:40) CD40 (BV711, 5C3, Biolegend, catalog number 334334, dilution 1:20), CD15 (W6D3, BV650, Biolegend, catalog number 323034, dilution 1:50), CD38 (HIT2, BV780, Becton Dickinson, catalog number 563964, dilution 1:40), CD86 (FUN1, BB515, Becton Dickinson, catalog number 564545, dilution 1:20), CD141 (M80, PerCP/Cy5.5, Biolegend, catalog number 344111, dilution 1:20). Dead cells were excluded with LIVE/DEAD® Fixable Aqua Dead Cell Stain Kit (Life Technologies Ltd). Cells where then fixed in 2% Paraformaldehyde (Electron Microscopy Sciences) and analyzed within 48 h from staining. Samples were acquired on a BD LSRFortessa™ X20. Data were analyzed by FlowJo (v10.5) using the gating strategy described in Supplementary Fig. 7.

### Reporting summary

Further information on research design is available in the Nature Research Reporting Summary linked to this article.

## Supplementary information

Supplementary Material

REPORTING SUMMARY

## Data Availability

The gene expression dataset generated and analyzed during the current study is available in the NCBI GEO repository, accession number GSE223316.

## Code availability

The underlying code for this study is not publicly available but may be made available to qualified researchers on reasonable request from the corresponding author.

## References

[1] Shang, M., Blanton, L., Brammer, L., Olsen, S. J. & Fry, A. M. Influenza-associated pediatric deaths in the United States, 2010-2016. *Pediatrics*10.1542/peds.2017-2918 (2018) 10.1542/peds.2017-2918 29440502

[2] Wong KK (2013). Influenza-associated pediatric deaths in the United States, 2004-2012. Pediatrics.

[3] Jefferson T, Rivetti A, Di Pietrantonj C, Demicheli V (2018). Vaccines for preventing influenza in healthy children. Cochrane Database Syst. Rev..

[4] Block SL (2012). Dose-range study of MF59-adjuvanted versus nonadjuvanted monovalent A/H1N1 pandemic influenza vaccine in six- to less than thirty-six-month-old children. Pediatr. Infect. Dis. J..

[5] Della Cioppa G, Vesikari T, Sokal E, Lindert K, Nicolay U (2011). Trivalent and quadrivalent MF59((R))-adjuvanted influenza vaccine in young children: a dose- and schedule-finding study. Vaccine.

[6] Nakaya HI (2016). Systems biology of immunity to MF59-adjuvanted versus nonadjuvanted trivalent seasonal influenza vaccines in early childhood. Proc. Natl Acad. Sci. USA.

[7] Puig-Barbera J, Perez-Vilar S, Diez-Domingo J (2011). MF59-adjuvanted seasonal influenza vaccine in young children. Expert Rev. Vaccines.

[8] Vesikari T (2012). Homologous and heterologous antibody responses to a one-year booster dose of an MF59((R)) adjuvanted A/H5N1 pre-pandemic influenza vaccine in pediatric subjects. Hum. Vaccines Immunother..

[9] Vesikari T, Groth N, Karvonen A, Borkowski A, Pellegrini M (2009). MF59-adjuvanted influenza vaccine (FLUAD) in children: safety and immunogenicity following a second year seasonal vaccination. Vaccine.

[10] Vesikari T (2009). Enhanced immunogenicity of seasonal influenza vaccines in young children using MF59 adjuvant. Pediatr. Infect. Dis. J..

[11] Patel SS, Bizjajeva S, Heijnen E, Oberye J (2019). MF59-adjuvanted seasonal trivalent inactivated influenza vaccine: Safety and immunogenicity in young children at risk of influenza complications. Int. J. Infect. Dis..

[12] Vesikari T (2011). Oil-in-water emulsion adjuvant with influenza vaccine in young children. N. Engl. J. Med..

[13] Patel SS, Bizjajeva S, Lindert K, Heijnen E, Oberye J (2019). Cumulative clinical experience with MF59-adjuvanted trivalent seasonal influenza vaccine in young children. Int. J. Infect. Dis..

[14] Zedda L (2015). Dissecting the immune response to MF59-adjuvanted and nonadjuvanted seasonal influenza vaccines in children less than three years of age. Pediatr. Infect. Dis. J..

[15] Liang, F. et al. Vaccine priming is restricted to draining lymph nodes and controlled by adjuvant-mediated antigen uptake. *Sci. Transl. Med.*10.1126/scitranslmed.aal2094 (2017). 10.1126/scitranslmed.aal2094 28592561

[16] Mosca F (2008). Molecular and cellular signatures of human vaccine adjuvants. Proc. Natl Acad. Sci. USA.

[17] Caproni E (2012). MF59 and Pam3CSK4 boost adaptive responses to influenza subunit vaccine through an IFN type I-independent mechanism of action. J. Immunol..

[18] Seubert A, Monaci E, Pizza M, O’Hagan DT, Wack A (2008). The adjuvants aluminum hydroxide and MF59 induce monocyte and granulocyte chemoattractants and enhance monocyte differentiation toward dendritic cells. J. Immunol..

[19] O’Hagan DT (2007). MF59 is a safe and potent vaccine adjuvant that enhances protection against influenza virus infection. Expert Rev. Vaccines.

[20] Fourati S (2022). Pan-vaccine analysis reveals innate immune endotypes predictive of antibody responses to vaccination. Nat. Immunol..

[21] Querec TD (2009). Systems biology approach predicts immunogenicity of the yellow fever vaccine in humans. Nat. Immunol..

[22] Gaucher D (2008). Yellow fever vaccine induces integrated multilineage and polyfunctional immune responses. J. Exp. Med..

[23] Li S (2014). Molecular signatures of antibody responses derived from a systems biology study of five human vaccines. Nat. Immunol..

[24] Li S (2017). Metabolic phenotypes of response to vaccination in humans. Cell.

[25] Qi Q (2016). Defective T memory cell differentiation after varicella zoster vaccination in older individuals. PLoS Pathog..

[26] Kazmin D (2017). Systems analysis of protective immune responses to RTS,S malaria vaccination in humans. Proc. Natl Acad. Sci. USA.

[27] van den Berg RA (2017). Predicting RTS,S vaccine-mediated protection from transcriptomes in a malaria-challenge clinical trial. Front. Immunol..

[28] Arunachalam PS (2021). Systems vaccinology of the BNT162b2 mRNA vaccine in humans. Nature.

[29] Nakaya HI (2011). Systems biology of vaccination for seasonal influenza in humans. Nat. Immunol..

[30] Nakaya HI (2015). Systems analysis of immunity to influenza vaccination across multiple years and in diverse populations reveals shared molecular signatures. Immunity.

[31] Furman, D. et al. Apoptosis and other immune biomarkers predict influenza vaccine responsiveness. *Mol. Syst. Biol.*10.1038/Msb.2013.15 (2013). 10.1038/msb.2013.15 PMC3658270 23591775

[32] Franco LM (2013). Integrative genomic analysis of the human immune response to influenza vaccination. Elife.

[33] Tsang JS (2014). Global analyses of human immune variation reveal baseline predictors of postvaccination responses. Cell.

[34] Cao RG (2014). Differences in antibody responses between trivalent inactivated influenza vaccine and live attenuated influenza vaccine correlate with the kinetics and magnitude of interferon signaling in children. J. Infect. Dis..

[35] Black S (2011). Hemagglutination inhibition antibody titers as a correlate of protection for inactivated influenza vaccines in children. Pediatr. Infect. Dis. J..

[36] Subramanian A (2005). Gene set enrichment analysis: a knowledge-based approach for interpreting genome-wide expression profiles. Proc. Natl Acad. Sci. USA.

[37] Blok BA, Arts RJ, van Crevel R, Benn CS, Netea MG (2015). Trained innate immunity as underlying mechanism for the long-term, nonspecific effects of vaccines. J. Leukoc. Biol..

[38] Netea MG, Latz E, Mills KH, O’Neill LA (2015). Innate immune memory: a paradigm shift in understanding host defense. Nat. Immunol..

[39] Netea MG (2013). Training innate immunity: the changing concept of immunological memory in innate host defence. Eur. J. Clin. Investig..

[40] Saeed S (2014). Epigenetic programming of monocyte-to-macrophage differentiation and trained innate immunity. Science.

[41] Wimmers F (2021). The single-cell epigenomic and transcriptional landscape of immunity to influenza vaccination. Cell.

[42] Lee A (2022). A molecular atlas of innate immunity to adjuvanted and live attenuated vaccines, in mice. Nat. Commun..

[43] Yamaguchi, Y. et al. Consecutive BNT162b2 mRNA vaccination induces short-term epigenetic memory in innate immune cells. *JCI Insight*10.1172/jci.insight.163347 (2022). 10.1172/jci.insight.163347 PMC9746816 36282593

[44] Silva ML (2011). Characterization of main cytokine sources from the innate and adaptive immune responses following primary 17DD yellow fever vaccination in adults. Vaccine.

[45] Martins MA (2008). Innate immunity phenotypic features point toward simultaneous raise of activation and modulation events following 17DD live attenuated yellow fever first-time vaccination. Vaccine.

[46] Neves PC, Matos DC, Marcovistz R, Galler R (2009). TLR expression and NK cell activation after human yellow fever vaccination. Vaccine.

[47] Mohanty S (2015). Prolonged proinflammatory cytokine production in monocytes modulated by interleukin 10 after influenza vaccination in older adults. J. Infect. Dis..

[48] Takano T (2022). Distinct immune cell dynamics correlate with the immunogenicity and reactogenicity of SARS-CoV-2 mRNA vaccine. Cell Rep. Med..

[49] Herve C (2019). The how’s and what’s of vaccine reactogenicity. NPJ Vaccines.

[50] Weiner J (2019). Characterization of potential biomarkers of reactogenicity of licensed antiviral vaccines: randomized controlled clinical trials conducted by the BIOVACSAFE consortium. Sci. Rep..

[51] Howard LM (2017). Cell-based systems biology analysis of human AS03-adjuvanted H5N1 avian influenza vaccine responses: a phase I randomized controlled trial. PLoS ONE.

[52] Burny W (2017). Different adjuvants induce common innate pathways that are associated with enhanced adaptive responses against a model antigen in humans. Front. Immunol..

[53] Kauffmann A, Gentleman R, Huber W (2009). arrayQualityMetrics–a bioconductor package for quality assessment of microarray data. Bioinformatics.

