# Supplementary material for: Memory-like innate response to booster vaccination with MF-59 adjuvanted influenza vaccine in children
Source: NPJ Vaccines. 2023 Jul 13;8:100. doi: 10.1038/s41541-023-00702-1 (PMC10344887; doi:10.1038/s41541-023-00702-1)
Supplement: Supplementary file 1 — Supplementary Material [file 41541_2023_702_MOESM1_ESM.pdf]

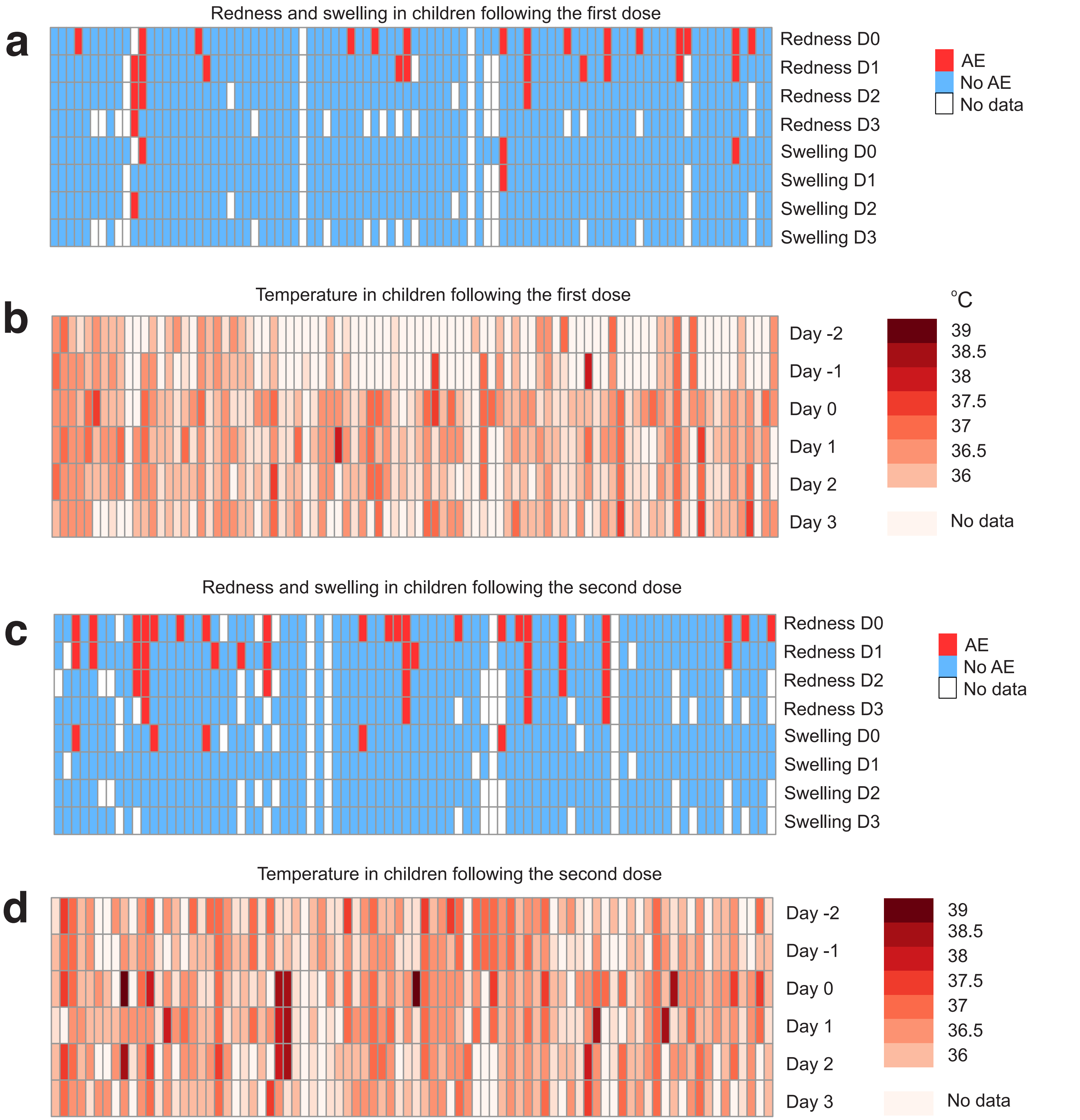

Supplementary Figure 1. Occurrence of adverse effects in children.
Each column represents a subject. An occurrence of an adverse effect is indicated by red color (panels A, C).
For fever (panels B, D) color represents the measured temperature.

a

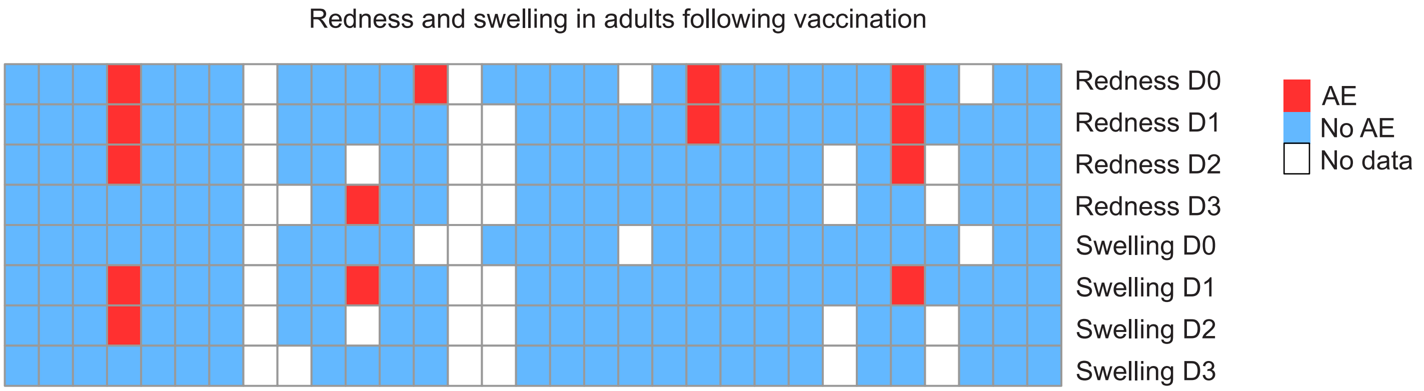

b

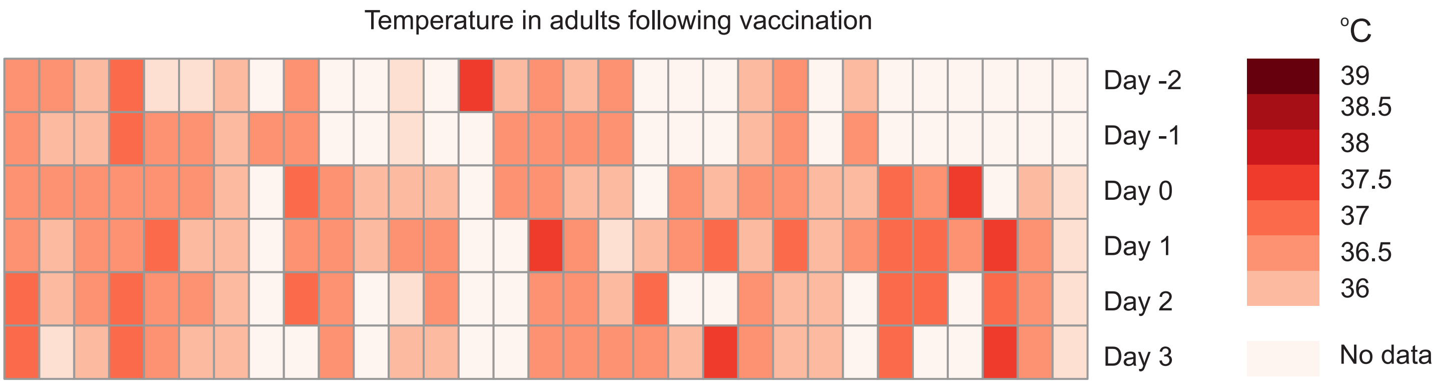

Supplementary Figure 2. Occurrence of adverse effects in adults.
Each column represents a subject. An occurrence of an adverse effect is indicated by red color (panel A).
For fever (panel B) color represents the measured temperature.

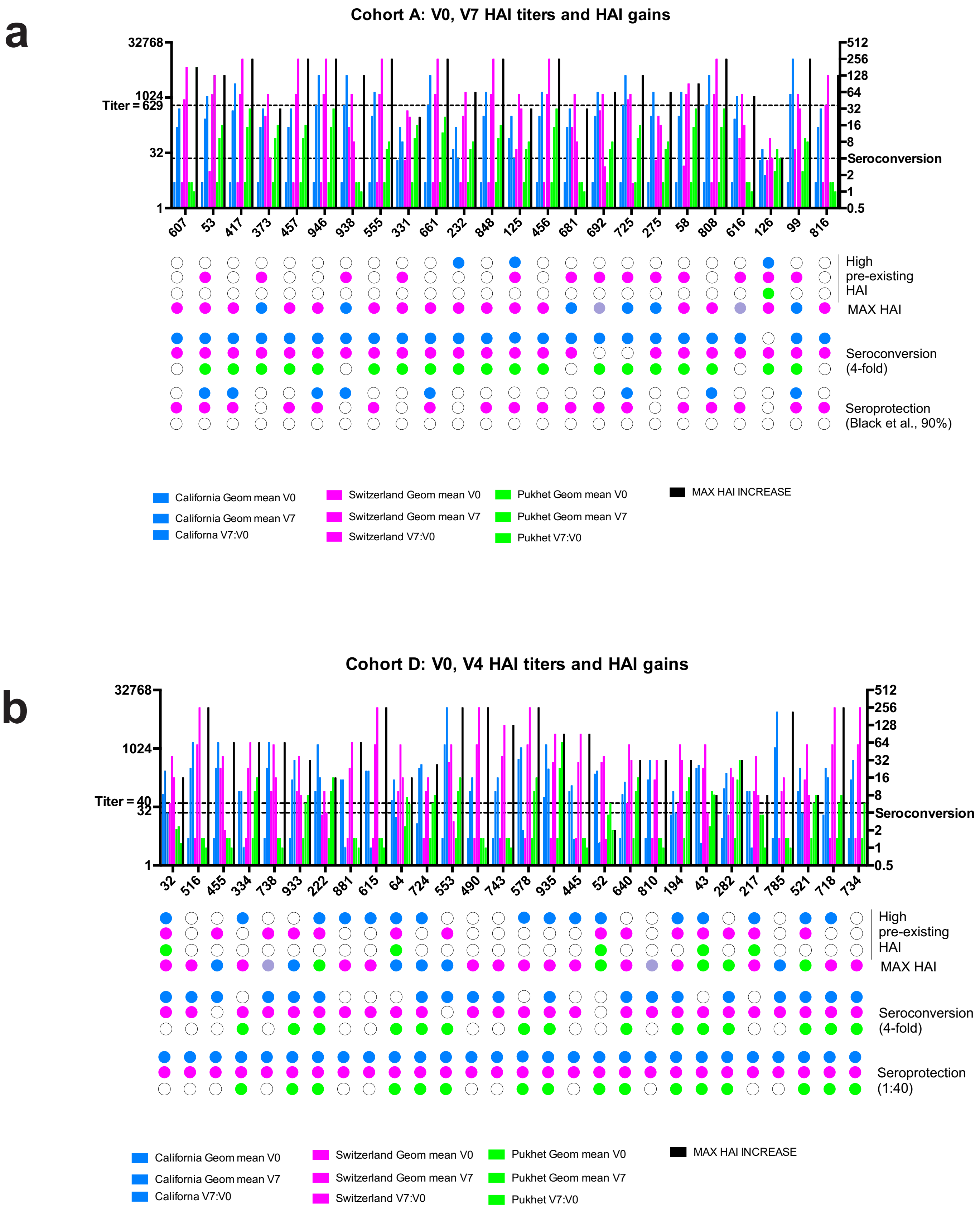
a
Cohort A: V0, V7 HAI titers and HAI gains
32768
1024
Titer = 629
32
1
512
256
128
64
32
16
8
Seroconversion
2
1
0.5
607 53 417 373 457 946 938 555 331 661 232 848 125 456 681 692 725 275 58 808 616 126 99 816
High pre-existing HAI
MAX HAI
Seroconversion (4-fold)
Seroprotection (Black et al., 90%)
California Geom mean V0
California Geom mean V7
Californa V7:V0
Switzerland Geom mean V0
Switzerland Geom mean V7
Switzerland V7:V0
Pukhet Geom mean V0
Pukhet Geom mean V7
Pukhet V7:V0
MAX HAI INCREASE
b
Cohort D: V0, V4 HAI titers and HAI gains
32768
1024
Titer = 40
32
1
512
256
128
64
32
16
8
Seroconversion
2
1
0.5
32 516 455 334 738 933 222 881 615 64 724 553 490 743 578 935 445 52 640 810 194 43 282 217 785 521 718 734
High pre-existing HAI
MAX HAI
Seroconversion (4-fold)
Seroprotection (1:40)
California Geom mean V0
California Geom mean V7
Californa V7:V0
Switzerland Geom mean V0
Switzerland Geom mean V7
Switzerland V7:V0
Pukhet Geom mean V0
Pukhet Geom mean V7
Pukhet V7:V0
MAX HAI INCREASE

Supplementary Figure 3. Pre-existing and final HAI titers per subject, seroconversion and seroprotection. A, Infant cohort A. B, Adult cohort D. For bar charts, each set of colored columns represents a subject. Within each set, there are groups of columns of four colors, each representing a vaccine strain or maximum gain in HAI titers across all three strains: blue: H1N1 (A/California), magenta: H3N2 (A/Switzerland), green: B/Phuket and black: MAX HAI gain. For each of the vaccine strains, there are three columns of the same color. The leftmost column (first) represents the HAI titer at the pre-vaccination baseline; the middle (second) column represents the HAI titer 28 days post final vaccination (day 56 of the study). These two columns relate to the left Y axis. The right (third) column represents the ratio of HAI titers at day 56 over day 0 (seroconversion) and should be read on the right Y axis. Black columns for MAX HAI gain also represent the change in HAI titers, and should be read on the right Y axis. For dot plots, each column of dots represents a subject, matching the order of subjects in the bar plot. Colored dots indicate whether the subject had a detectable pre-existing HAI titer against a given strain (first set of dots); whether seroconversion (4-fold increase in HAI titers over the baseline) was achieved against a particular strain (second set of dots); and whether seroprotection was achieved against a particular strain (third set of dots). Dots are color coded according to the strain as in the bar plot. The row of dots labeled “MAX HAI” indicates against which strain the maximum gain in HAI titers was achieved.

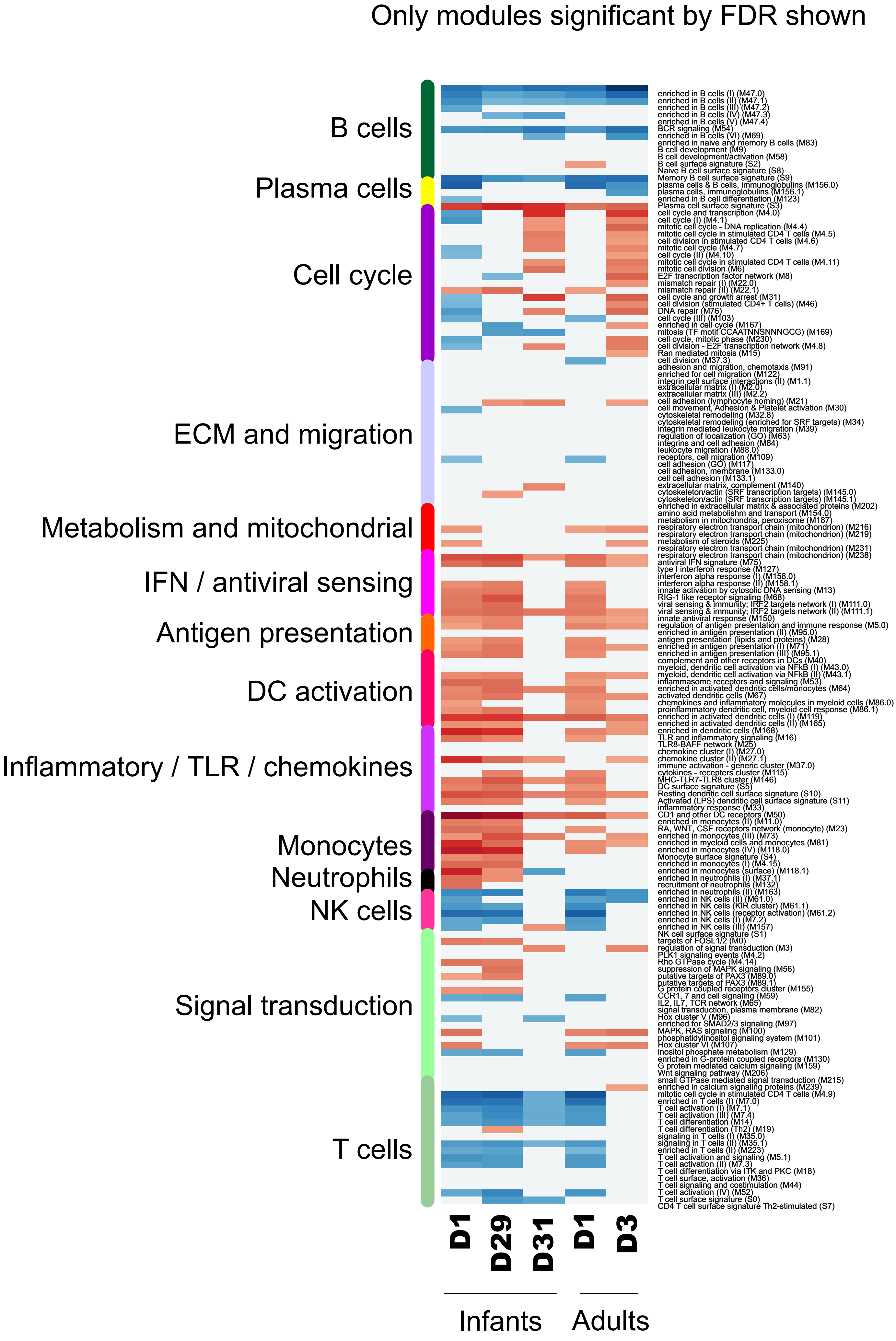

Only modules significant by FDR shown
B cells
Plasma cells
Cell cycle
ECM and migration
Metabolism and mitochondrial
IFN / antiviral sensing
Antigen presentation
DC activation
Inflammatory / TLR / chemokines
Monocytes
Neutrophils
NK cells
Signal transduction
T cells
enriched in B cells (I) (M47.0)
enriched in B cells (II) (M47.1)
enriched in B cells (III) (M47.2)
enriched in B cells (IV) (M47.3)
enriched in B cells (V) (M47.4)
BCR signaling (M54)
enriched in B cells (VI) (M69)
enriched in naive and memory B cells (M83)
B cell development (M9)
B cell development/activation (M58)
B cell surface signature (S2)
Naive B cell surface signature (S8)
Memory B cell surface signature (S9)
plasma cells & B cells, immunoglobulins (M156.0)
plasma cells, immunoglobulins (M156.1)
enriched in B cell differentiation (M123)
Plasma cell surface signature (S3)
cell cycle and transcription (M4.0)
cell cycle (I) (M4.1)
mitotic cell cycle - DNA replication (M4.4)
mitotic cell cycle in stimulated CD4 T cells (M4.5)
cell division in stimulated CD4 T cells (M4.6)
mitotic cell cycle (M4.7)
cell cycle (II) (M4.10)
mitotic cell cycle in stimulated CD4 T cells (M4.11)
mitotic cell division (M6)
E2F transcription factor network (M8)
mismatch repair (I) (M22.0)
mismatch repair (II) (M22.1)
cell cycle and growth arrest (M31)
cell division (stimulated CD4+ T cells) (M46)
DNA repair (M76)
cell cycle (III) (M103)
enriched in cell cycle (M167)
mitosis (TF motif CCAATNNSNNNGCG) (M169)
cell cycle, mitotic phase (M230)
cell division - E2F transcription network (M4.8)
Ran mediated mitosis (M15)
cell division (M37.3)
adhesion and migration, chemotaxis (M91)
enriched for cell migration (M122)
integrin cell surface interactions (II) (M1.1)
extracellular matrix (I) (M2.0)
extracellular matrix (III) (M2.2)
cell adhesion (lymphocyte homing) (M21)
cell movement, Adhesion & Platelet activation (M30)
cytoskeletal remodeling (M32.8)
cytoskeletal remodeling (enriched for SRF targets) (M34)
integrin mediated leukocyte migration (M39)
regulation of localization (GO) (M63)
integrins and cell adhesion (M84)
leukocyte migration (M88.0)
receptors, cell migration (M109)
cell adhesion (GO) (M117)
cell adhesion, membrane (M133.0)
cell cell adhesion (M133.1)
extracellular matrix, complement (M140)
cytoskeleton/actin (SRF transcription targets) (M145.0)
cytoskeleton/actin (SRF transcription targets) (M145.1)
enriched in extracellular matrix & associated proteins (M202)
amino acid metabolism and transport (M154.0)
metabolism in mitochondria, peroxisome (M187)
respiratory electron transport chain (mitochondrion) (M216)
respiratory electron transport chain (mitochondrion) (M219)
metabolism of steroids (M225)
respiratory electron transport chain (mitochondrion) (M231)
respiratory electron transport chain (mitochondrion) (M238)
antiviral IFN signature (M75)
type I interferon response (M127)
interferon alpha response (I) (M158.0)
interferon alpha response (II) (M158.1)
innate activation by cytosolic DNA sensing (M13)
RIG-1 like receptor signaling (M68)
viral sensing & immunity; IRF2 targets network (I) (M111.0)
viral sensing & immunity; IRF2 targets network (II) (M111.1)
innate antiviral response (M150)
regulation of antigen presentation and immune response (M5.0)
enriched in antigen presentation (II) (M95.0)
antigen presentation (lipids and proteins) (M28)
enriched in antigen presentation (I) (M71)
enriched in antigen presentation (III) (M95.1)
complement and other receptors in DCs (M40)
myeloid, dendritic cell activation via NFkB (I) (M43.0)
myeloid, dendritic cell activation via NFkB (II) (M43.1)
inflammasome receptors and signaling (M53)
enriched in activated dendritic cells/monocytes (M64)
activated dendritic cells (M67)
chemokines and inflammatory molecules in myeloid cells (M86.0)
proinflammatory dendritic cell, myeloid cell response (M86.1)
enriched in activated dendritic cells (I) (M119)
enriched in activated dendritic cells (II) (M165)
enriched in dendritic cells (M168)
TLR and inflammatory signaling (M16)
TLR8-BAFF network (M25)
chemokine cluster (I) (M27.0)
chemokine cluster (II) (M27.1)
immune activation - generic cluster (M37.0)
cytokines - recepters cluster (M115)
MHC-TLR7-TLR8 cluster (M146)
DC surface signature (S5)
Resting dendritic cell surface signature (S10)
Activated (LPS) dendritic cell surface signature (S11)
inflammatory response (M33)
CD1 and other DC receptors (M50)
enriched in monocytes (II) (M11.0)
RA, WNT, CSF receptors network (monocyte) (M23)
enriched in monocytes (III) (M73)
enriched in myeloid cells and monocytes (M81)
enriched in monocytes (IV) (M118.0)
Monocyte surface signature (S4)
enriched in monocytes (I) (M4.15)
enriched in monocytes (surface) (M118.1)
enriched in neutrophils (I) (M37.1)
recruitment of neutrophils (M132)
enriched in neutrophils (II) (M163)
enriched in NK cells (II) (M61.0)
enriched in NK cells (KIR cluster) (M61.1)
enriched in NK cells (receptor activation) (M61.2)
enriched in NK cells (I) (M7.2)
enriched in NK cells (III) (M157)
NK cell surface signature (S1)
targets of FOSL1/2 (M0)
regulation of signal transduction (M3)
PLK1 signaling events (M4.2)
Rho GTPase cycle (M4.14)
suppression of MAPK signaling (M56)
putative targets of PAX3 (M89.0)
putative targets of PAX3 (M89.1)
G protein coupled receptors cluster (M155)
CCR1, 7 and cell signaling (M59)
IL2, IL7, TCR network (M65)
signal transduction, plasma membrane (M82)
Hox cluster V (M96)
enriched for SMAD2/3 signaling (M97)
MAPK, RAS signaling (M100)
phosphatidylinositol signaling system (M101)
Hox cluster VI (M107)
inositol phosphate metabolism (M129)
enriched in G-protein coupled receptors (M130)
G protein mediated calcium signaling (M159)
Wnt signaling pathway (M206)
small GTPase mediated signal transduction (M215)
enriched in calcium signaling proteins (M239)
mitotic cell cycle in stimulated CD4 T cells (M4.9)
enriched in T cells (I) (M7.0)
T cell activation (I) (M7.1)
T cell activation (III) (M7.4)
T cell differentiation (M14)
T cell differentiation (Th2) (M19)
signaling in T cells (I) (M35.0)
signaling in T cells (II) (M35.1)
enriched in T cells (II) (M223)
T cell activation and signaling (M5.1)
T cell activation (II) (M7.3)
T cell differentiation via ITK and PKC (M18)
T cell surface, activation (M36)
T cell signaling and costimulation (M44)
T cell activation (IV) (M52)
T cell surface signature (S0)
CD4 T cell surface signature Th2-stimulated (S7)
D1
D29
D31
D1
D3
Infants
Adults

Supplementary Figure 4. Significantly regulated gene modules in response to vaccination. Same as Figure 3B, but all includes all modules regardless of significance.

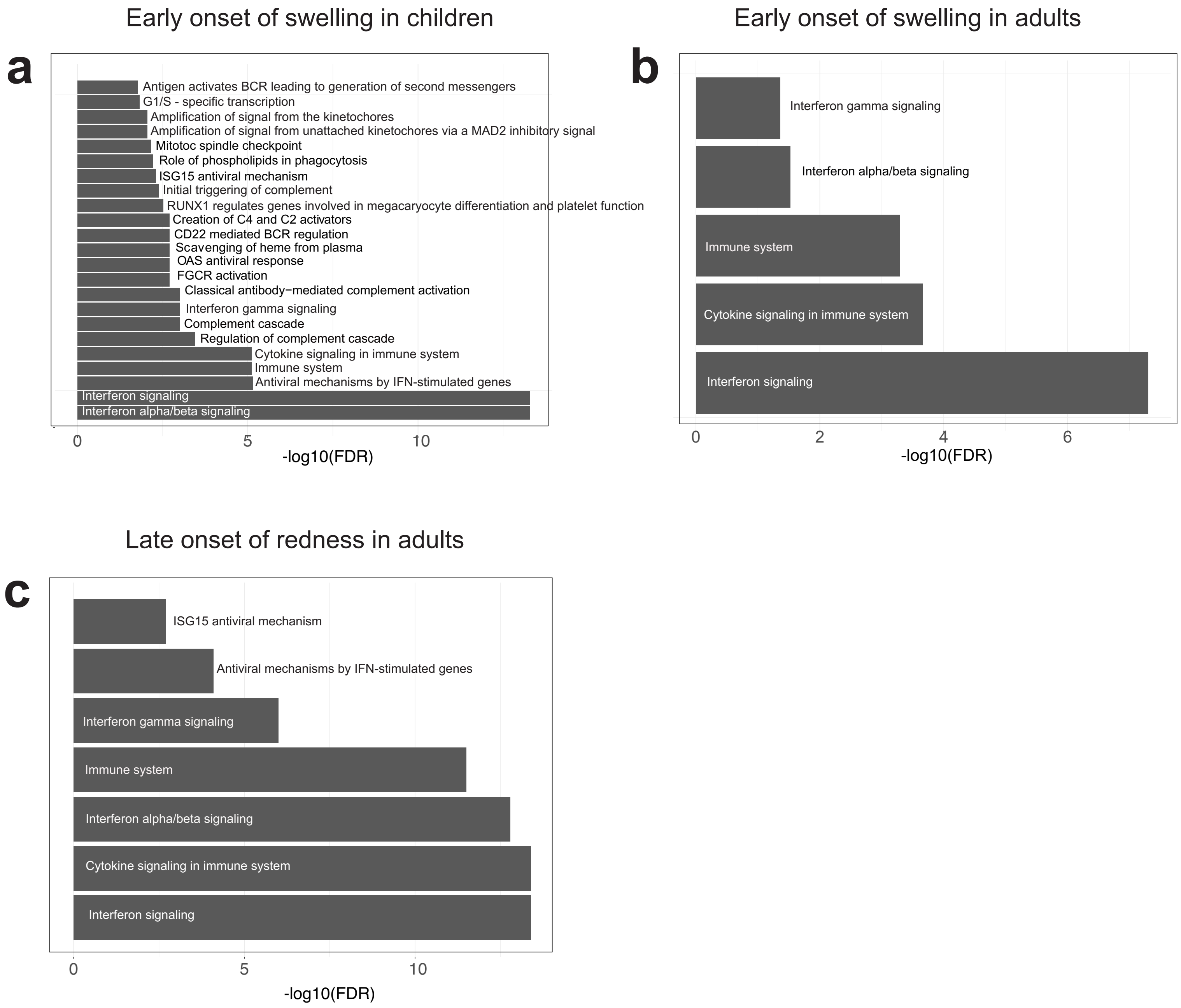

Supplementary Figure 5. Transcriptional correlates of reactogenicity post-vaccination.
Same as Figure 5B, showing significantly enriched pathways that are upregulated in responders for early onset of swelling in children (A), early onset of swelling in adults (B) and late onset of erythema in adults (C).

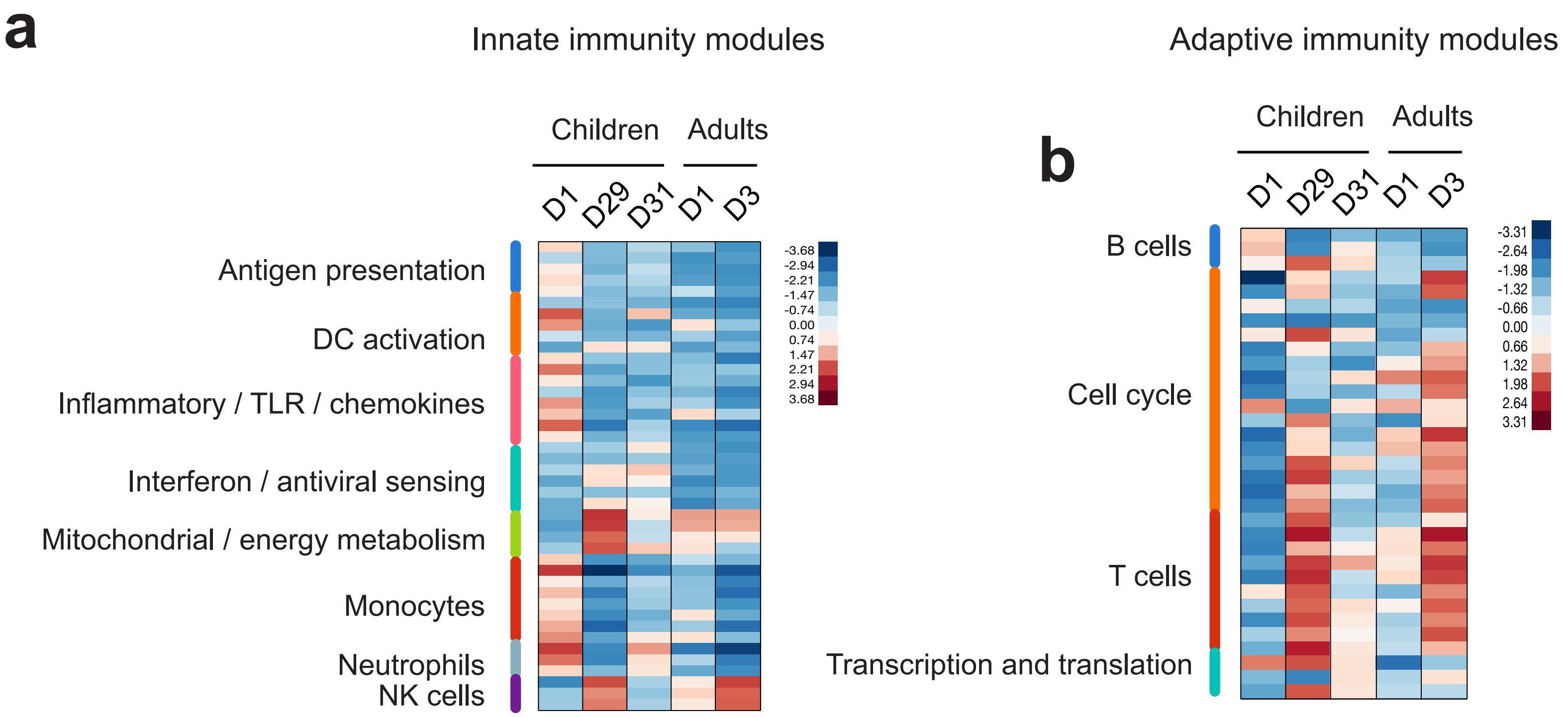

Supplementary Figure 6. Transcriptional correlates of immunogenicity.
A, B, Same as Figure 6BC, but with all modules shown, regardless of significance.

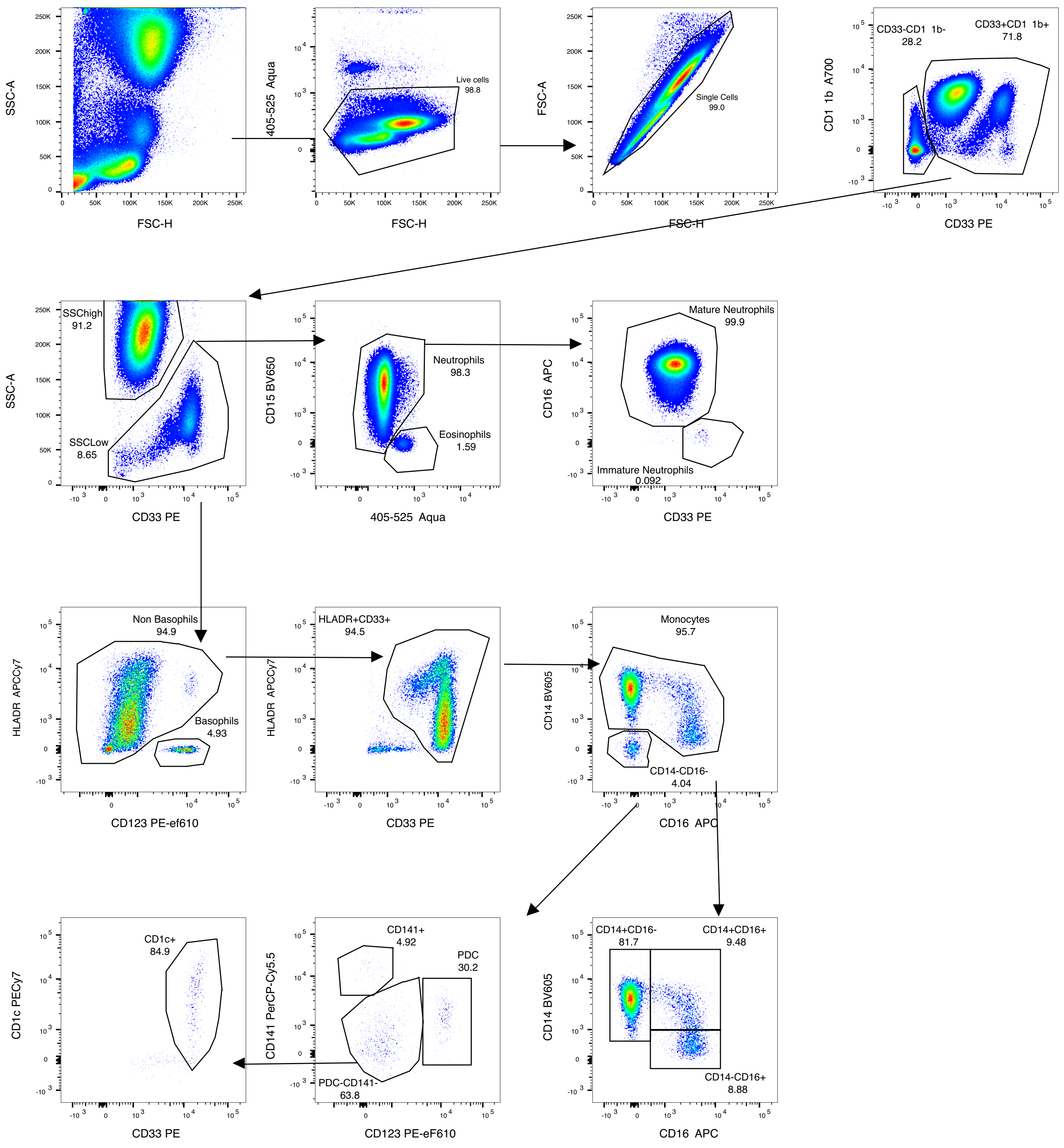

Supplementary Figure 7. Gating strategy for immunoprofiling of innate cellular subsets.
